# Fibrodysplasia Ossificans Progressiva: A Case Report

**DOI:** 10.7759/cureus.55528

**Published:** 2024-03-04

**Authors:** Linzeng Qi, Yongyuan Guo

**Affiliations:** 1 Orthopedics, Qilu Hospital of Shandong University, Jinan, CHN

**Keywords:** congenital great toe malformation, treatment strategies, genetic mutation, ectopic ossification, fibrodysplasia ossificans progressiva (fop)

## Abstract

Fibrodysplasia ossificans progressiva (FOP) is a rare autosomal dominant genetic disorder characterized by congenital great toe malformations and progressive ectopic ossification. We report a typical case of FOP in a 22-year-old female patient presenting with limited movement of the left knee joint, which began following trauma in 2019. Clinical examination revealed a large mass behind the left knee, bilateral great toe deformities, and no palpable superficial lymph nodes, without systemic pain or other discomfort. Imaging and genetic testing further supported the diagnosis of FOP, demonstrating high-density ossification within soft tissues and a mutation in the *ACVR1* gene. Treatment involved a combination of methylprednisolone and alendronate sodium vitamin D3 tablets, which yielded some therapeutic efficacy. The discussion emphasizes clinical diagnosis, pathogenesis, and treatment strategies for FOP, including injury prevention, rehabilitation exercises, and pharmacological interventions. Despite the lack of definitive treatment options, timely diagnosis and comprehensive management can effectively alleviate symptoms and improve the quality of life for affected individuals.

## Introduction

Fibrodysplasia ossificans progressiva (FOP), also known as myositis ossificans progressiva (MOP), is a rare autosomal dominant genetic disorder characterized by congenital malformation of the great toes and progressive ectopic ossification, with an estimated incidence of 1/2,000,000 to 1/1,300,000 [1]. Clinically, it is mostly reported as individual cases, with limited research on its pathogenesis and treatment strategies [2]. Due to the challenging clinical course of this patient, we report a typical case of FOP and provide an analysis and summary of its clinical characteristics and treatment.

## Case presentation

**Figure 1:**
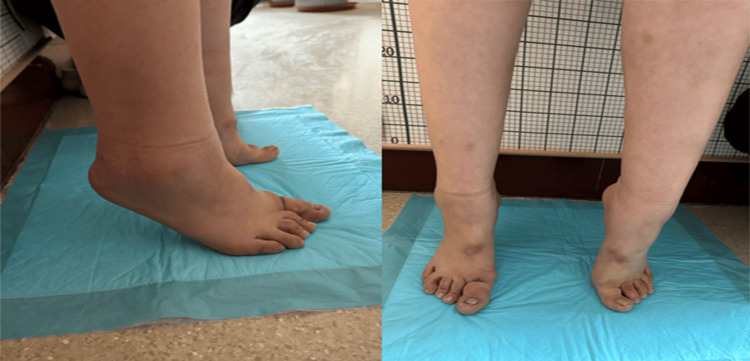
Photographs of the anteroposterior and lateral ankle joints Double full arch disappeared, great hallux valgus deformity.

A 22-year-old female patient was admitted to the Department of Orthopedics at Qilu Hospital of Shandong University on December 3, 2023, due to limited movement of the left knee joint for three years. Since sustaining trauma in May 2019, followed by a 15-day hospitalization at a local hospital, the patient experienced abnormal movement in both feet, left knee, left hip, and right shoulder. Over the subsequent three years, coinciding with the COVID-19 pandemic, the patient's symptoms worsened progressively despite infection with the virus and vaccination. Presently, the patient has lost mobility in the lumbar spine, cervical spine, and left knee, with a large mass behind the left knee originating from the distal femur. Mobility is also compromised in the right shoulder. Muscle strength and sensation in all limbs are normal. The patient exhibits bilateral great toe deformities characterized by outward deviation (Figure 1).

**Figure 2:**
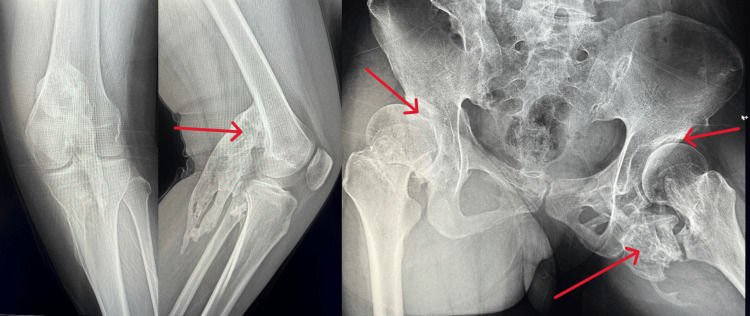
The X-ray of the left knee and hip There was a large mass in the left knee joint, and dysplasia and multiple large masses in the hip joint.

Laboratory investigations including blood routine, inflammatory markers, thyroid function, anti-nuclear antibodies/antibody spectrum, markers of vasculitis, IgG4 levels, and tumor markers were within normal limits. Alkaline phosphatase (ALP) levels showed no significant abnormalities. Whole-body long bone radiography revealed high-density ossification in the soft tissue behind the left knee joint and irregular bone density along the inner edge of the upper segment of the right tibia. The spinal curvature was straightened, with irregular morphology of multiple ribs. Bilateral hip dysplasia and osteoarthritis were noted, along with patchy ossification below the right hip joint (Figure 2).

**Figure 3:**
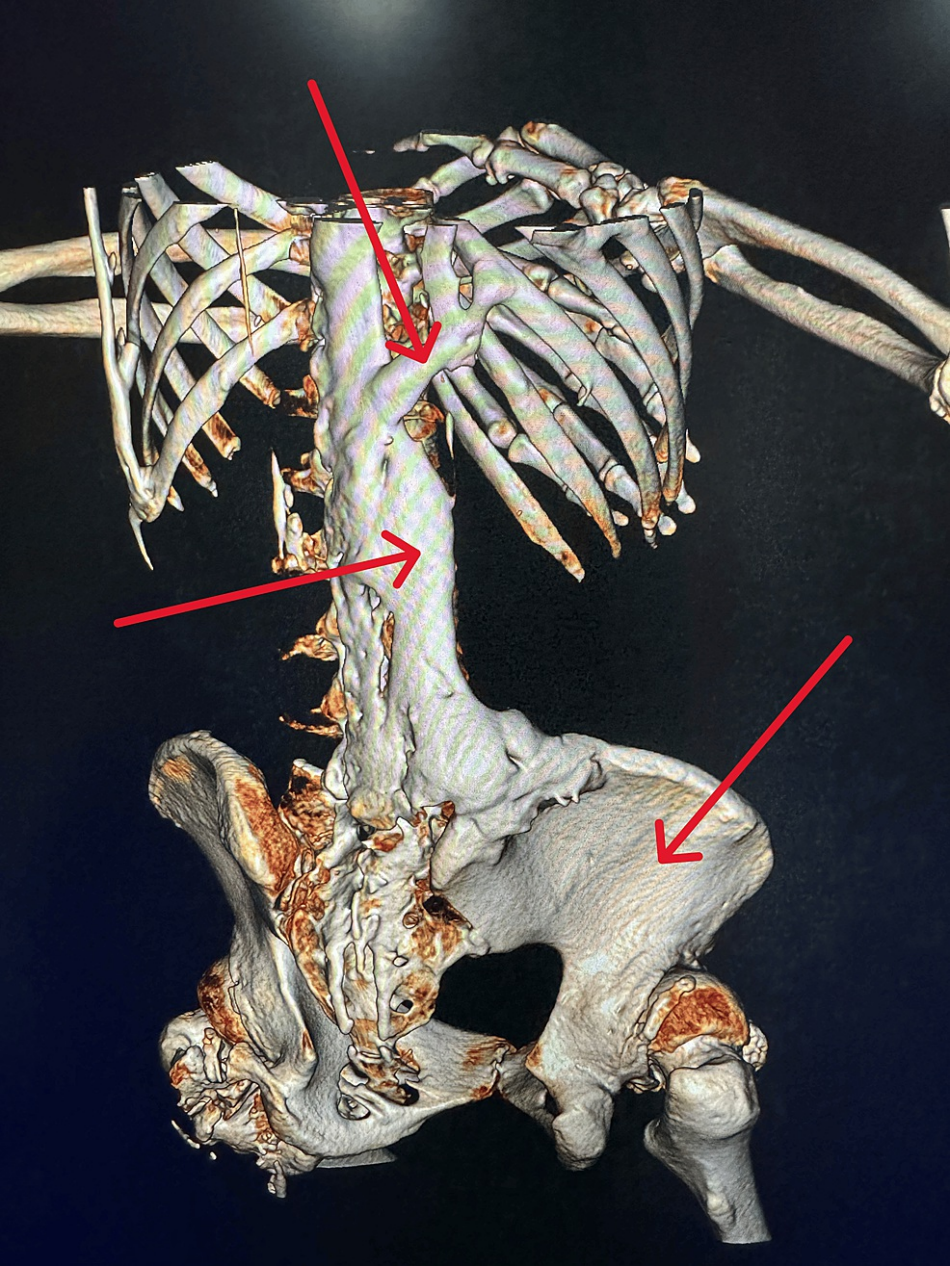
CT imaging Multiple ectopic ossification can be seen throughout the body.

Whole-body CT revealed no significant abnormalities in the skull, with partial fusion of cervical spinous processes and posterior edges. Ectopic ossification was observed around the bilateral scapulae, hip joints, knee joints, and lumbar spine with the ilium (Figure 3).

**Figure 4:**
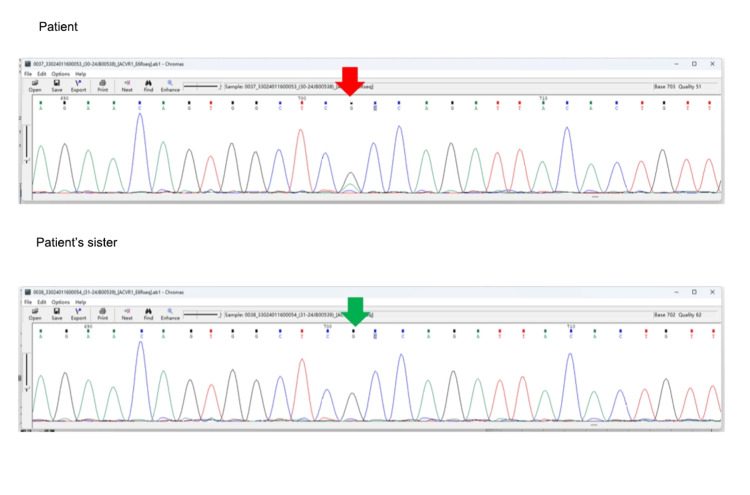
Genetic test results The mutation site of the gene.

Genetic testing using whole-exome sequencing and copy number analysis revealed a mutation in *ACVR1* (NM-001105.4), with a heterozygous pathogenic variant at c.617G>A (p.Arg206His) (Figure 4).

The patient's 12-year-old sister had a wild-type *ACVR1* genotype. Additionally, mutations in ACAN, LIFR, and HSPG2 genes were observed. Based on the comprehensive clinical history and multidisciplinary discussions between orthopedics and radiology, the patient was diagnosed with FOP. The patient did not report systemic pain or other discomfort, and her condition remained stable. Treatment included intravenous administration of methylprednisolone 40 mg once daily and oral alendronate sodium vitamin D3 tablets 70.14 mg once weekly to suppress ectopic ossification. The patient was discharged with instructions for regular follow-up appointments.

## Discussion

FOP is a connective tissue disorder characterized by congenital great toe abnormalities and progressive ectopic ossification. It is a rare, debilitating genetic disease with no specific gender, racial, or geographic predilection [2,3]. Early manifestations are intermittent, gradually progressing from the cervical, thoracic, and dorsal regions to the proximal ends of the limbs. The median lifespan in FOP patients is around 40 years, primarily due to severe infections or multiorgan failure resulting from diaphragmatic involvement [4]. Diagnosis relies mainly on clinical criteria, including congenital great toe malformations, progressive ectopic ossification, and anatomical changes during disease progression [5]. Enhanced CT or MRI can detect ectopic ossification and structural alterations, facilitating diagnosis. In this case, a 22-year-old female patient presented with stable acupuncture stimulation [6,7]. The pathogenesis of FOP primarily involves the bone morphogenetic protein receptor *ACVR1*, with mutations occurring in approximately 95% of FOP patients, most commonly the exon c.617G>A (p.Arg206His) mutation, leading to classical clinical manifestations. In this case, genetic analysis revealed a de novo heterozygous missense mutation in *ACVR1* at c.617G>A (p.Arg206His), with wild-type genotypes observed in the patient's sister. Additionally, the patient exhibited multiple osteochondromas and multiple ectopic ossifications, highlighting the importance of screening for congenital malformations and benign soft tissue tumors in clinical FOP patients. FOP can be misdiagnosed as dermatomyositis, myositis ossificans, osteochondromatosis, or myofibroblastic tumors; therefore, combining imaging and genetic diagnostics can reduce clinical misdiagnosis rates.

Current treatment strategies for FOP emphasize early diagnosis, injury prevention, and rehabilitation exercises [8-10]. This includes avoiding soft tissue and muscle injuries and minimizing local stimulation. Appropriate rehabilitation therapy aims to improve lung function, enhance muscle strength, and increase joint mobility, thereby improving the quality of life in the later stages of the disease. Pharmacological treatments include short-term symptomatic treatments such as non-steroidal anti-inflammatory drugs, mast cell inhibitors, glucocorticoids, and immunosuppressants during early acute inflammatory reactions. However, long-term and high-dose use is not recommended due to potential adverse effects. During the fibroproliferative phase, anti-angiogenic drugs and endothelial growth factor inhibitors may be used. In the ossification formation phase, abnormal expression of bone morphogenetic protein-4 leads to cartilage formation and subsequent ectopic calcification. Therefore, inhibitors of bone morphogenetic protein-4, amino bisphosphonates, and bisphosphonates may have certain therapeutic effects [11].

Emerging therapies targeting *ACVR1* include inhibitors of hypoxia-inducible factor-1α for inhibiting ectopic ossification, *ACVR1* kinase inhibitors (LDN 212454) for reducing ectopic ossification, and activin A neutralizing antibodies for effectively suppressing ectopic ossification, offering the potential for developing highly selective and specific drugs for FOP treatment. However, it should be emphasized that these measures only delay or alleviate the progression of the disease and do not effectively halt or reverse its progression. Some researchers suggest that low-dose graded radiation therapy for early ectopic ossification may be effective, while others propose that retinoid agonists and fluoroquinolones may effectively suppress ectopic ossification. In terms of gene therapy for FOP, research is progressing in several directions, but it has not yet reached the clinical application stage. There are some advances in gene therapy and the potential for future treatments.

Gene editing technology

Using gene editing techniques such as CRISPR-Cas9, researchers are attempting to correct mutations in the *ACVR1* gene in cells of FOP patients to restore normal receptor function. This approach has the potential to directly target the disease-causing genes for repair, but it still faces many challenges, such as ensuring the accuracy and safety of the repair [12,13].

RNA interference (RNAi)

RNAi technology can inhibit the expression of the *ACVR1* gene by targeting RNA molecules, thereby reducing abnormal ossification. Researchers have demonstrated the inhibitory effect of RNAi on the pathological process of FOP in animal models, but further research is needed to determine its effectiveness and safety in humans [14].

Gene therapy vectors

Using viruses or other vectors to introduce normal *ACVR1* genes into patients' cells to restore receptor function. While this approach has been applied in other gene therapy fields, it is still in the early stages for FOP treatment and requires overcoming safety and efficacy issues associated with gene therapy [15].

Stem cell therapy

Utilizing stem cell technology, researchers are attempting to repair receptor function by implanting normal *ACVR1* genes and promoting the regeneration and repair of receptor cells. Although this approach holds promise, it still needs to address safety issues such as transplant rejection and tumor formation [16]. In conclusion, gene therapy holds great potential for the treatment of FOP, but it still faces many challenges. Future research will continue to explore various gene therapy strategies and strive to overcome technical and clinical obstacles to provide more effective treatment options for FOP patients. In summary, there are currently no definitive effective treatment modalities for FOP. 

## Conclusions

This study presents a typical case of FOP, providing a detailed description and analysis of its clinical presentation, imaging features, and genetic testing results. The patient exhibited typical symptoms of FOP following trauma, including progressive joint restriction, toe malformation, and ectopic ossification within soft tissues. Radiographic examination revealed corresponding ossification images, while genetic testing further confirmed a mutation in the *ACVR1* gene, supporting the diagnosis of FOP. Treatment with methylprednisolone and alendronate sodium vitamin D3 tablets showed some therapeutic efficacy. The discussion emphasizes clinical diagnosis and treatment strategies for FOP, including injury prevention, rehabilitation exercises, and pharmacological interventions. Despite the lack of effective treatment modalities currently available, this study provides important clinical insights for the early diagnosis and comprehensive management of FOP, aiming to improve patient quality of life and delay disease progression. Further large-scale clinical research is warranted to explore more effective treatment approaches and provide better medical care and support for patients with FOP.
